# Acupuncture for patients with mild hypertension: study protocol of an open-label multicenter randomized controlled trial

**DOI:** 10.1186/1745-6215-14-380

**Published:** 2013-11-11

**Authors:** Juan Li, Hui Zheng, Ling Zhao, Ying Li, Yan Zhang, Xiao-rong Chang, Rui-hui Wang, Jing Shi, Jin Cui, Yin-lan Huang, Xiang Li, Jie Chen, De-hua Li, Fan-rong Liang

**Affiliations:** 1 Chengdu University of Traditional Chinese Medicine Chengdu, Chengdu, Sichuan, PR China; 2 First Affiliated Hospital, Chengdu University of Traditional Chinese Medicine, Chengdu, Sichuan, PR China; 3 Hunan University of Traditional Chinese Medicine, Changsha, Hunan, PR China; 4 Shanxi College of Traditional Chinese Medicine, Xi’an, Shanxi, PR China; 5 Yunnan Provincial Hospital of Traditional Chinese Medicine, Kunming, Yunnan, PR China; 6 Guiyang College of Traditional Chinese Medicine, Guiyang, Guizhou, PR China; 7 Ningxia Medical University, Ningxia, Gansu, PR China

**Keywords:** Essential hypertension, Acupuncture, Acupoint specificity, Acupoints effect, Randomized controlled trials, Protocol

## Abstract

**Background:**

Several studies using acupuncture to treat essential hypertension have been carried out. However, whether acupuncture is efficacious for hypertension is still controversial. Therefore, this trial aims to evaluate the efficacy and safety of acupuncture for patients with mild hypertension.

**Methods/Design:**

This is a large scale, open-label, multicenter, randomized controlled clinical trial with four parallel arms. We will recruit 428 hypertensive patients with systolic blood pressure (SBP) between 140 and 159 mmHg, diastolic blood pressure (DBP) between 90 and 99 mmHg. The participants will be randomly assigned to four different groups (three acupuncture groups and one waiting list group) (1).The affected meridian acupuncture group (*n* = 107) is treated with acupoints on the affected meridians (2).The non-affected meridian acupuncture group (*n* = 107) is treated with acupoints on the non-affected meridians (3).The invasive sham acupuncture group (*n* = 107) is provided with sham acupoints treatment (4).The waiting-list group (*n* = 107) is not offered any intervention until they complete the trial. Each patient allocated to acupuncture groups will receive 18 sessions of acupuncture treatment over 6 weeks. This trial will be conducted in 11 hospitals in China. The primary endpoint is the change in average 24-hSBP before and 6 weeks after randomization. The secondary endpoints are average SBP and average DBP during the daytime and night-time, and 36-Item Short Form Survey (SF-36), and so on.

**Discussion:**

This is the first large scale, multicenter, randomized, sham controlled trial of acupuncture for essential hypertension in China. It may clarify the efficacy of acupuncture as a treatment for mild hypertension.

**Trial registration:**

Clinicaltrials.gov Identifier: NCT01701726

## Background

Essential hypertension continues to be an important public-health challenge worldwide [1]. Hypertension is an independent risk factor for premature death, heart disease, stroke, peripheral vascular disease, and renal failure. Hypertension affects 29.3% of the adult population in the United States [2]. In 2009,the direct and indirect costs for hypertension in the United States are projected to be approximately $73.4 billion [3]. In the fourth National Nutrition and Health Survey in 2002, the prevalence of hypertension among Chinese population was 18.8% [4], affecting approximately 200 million individuals in China, while mild hypertension accounts for >60% [5]. Furthermore, the number of hypertensive patients is still increasing [6]. It is also reported that in China, the direct medical costs for hypertensive patientsaged 35 to 75 years are ¥20.2 billion [7].

Pharmacological treatment remains the chief treatment option for mild hypertension. However, a recent Cochrane review found that there is no proved benefit in antihypertensive drugs treatment for patients with mild hypertension. In addition, owing to adverse effects of antihypertensive drugs, 9% of patients discontinue their treatment [8]. Non-pharmacological interventions including acupuncture prevail as a treatment option for hypertensive patients [9]. In China, acupuncture has been used to treat disease for >2,500 years, and it is also widely used all around the world. Nowadays, acupuncture is used to treat cardiovascular disease (such as angina pectoris [10], hypertension [11]). Several randomized controlled trials of acupuncture to treat hypertension had been conducted, but the results were not consistent. Therefore, we designed a multicenter randomized controlled trial to confirm whether acupuncture is efficacious and safe for hypertension.

In this trial, we first intend to investigate the efficacy of acupuncture to lower the blood pressure comparing the waiting-list control and invasive sham acupuncture groups. Second, we attempt to determine whether there is a difference between acupoints on the affected meridians according to meridian syndrome differentiation and the acupoints on the non-affected meridians. This clinical trial is financed by the national basic research program (973program) in China, and is registered with an identifier (http://NCT01701726) by clinicaltrials.gov in the USA.

## Methods/Design

### Design

This trial is an open-label multicenter randomized controlled trial with four parallel arms. We compare the affected meridians with non-affected meridians, sham acupuncture and waiting-list groups as well. The trial is performed in the following Hospitals: First Affiliated Hospital of Chengdu University of Traditional Chinese Medicine; Third Affiliated Hospital of Chengdu University of Traditional Chinese Medicine (TCM); Affiliated Hospital of Ningxia Medicine University; First Affiliated Hospital of Hunan University of TCM; Second Affiliated Hospital of Hunan University of TCM; Affiliated Hospital of Hunan University of TCM - Hengyang Hospital; Chenzhou No.1 People’s Hospital; First Affiliated Hospital of Guiyang College of TCM; Second Affiliated Hospital of Guiyang College of TCM; Yunnan Provincial Hospital of TCM; and Second Affiliated Hospital of Shanxi College of TCM.

**Figure 1:**
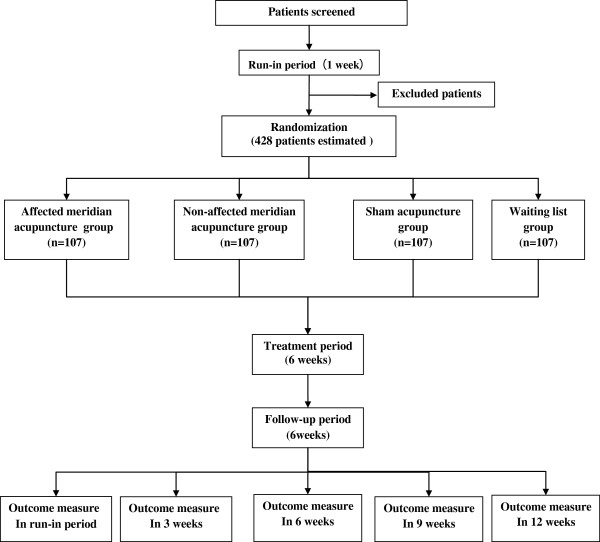
Trial flow chart.

The whole study period is 13 weeks,with a 1-week run-in period, 6-week treatment phase, and 6-week follow-up phase. After randomization, patients in acupuncture groups will receive 18 sessions of treatment over a 6-week treatment period. Participants in the waiting-list control group will give an option to receive acupuncture treatment after 13 weeks study period. Outcome measurements will be assessed at day 0, 6 weeks, 9 weeks, and 12 weeks after randomization. All patients will be informed that they may be randomly allocated to affected meridian acupuncture group, non-affected acupuncture group, invasive sham acupuncture group, or waiting-list group (Figure 1, Table 1). This trial will be conducted according to the principles of the Declaration of Helsinki (version Edinburgh 2000). The trial protocol has been approved by the regional ethical review committee of traditional Chinese medicine in Sichuan Province on April 2012. We will require all patients to sign the written informed consent, and they will be given enough time to decide whether they will take part in this trial. This trial started in September 2012, and will be completed in December 2017.

**Table 1:** Time to visit and data collection

	**-1 week**	**0 week**	**3 weeks**	**6 weeks**	**3 weeks after treatment**	**6 weeks after treatment**
	Baseline	Treatment phase			Follow-up phase	
**Patients**	X					
Informed consent	x					
Medical history	x					
Physical examination		x				
Randomization						
**Intervention**						
Affected meridian acupuncture group (*n* = 107)		18 sessions of acupuncture at acupoints				
Non-affected meridian acupuncture group (*n* = 107)						
**Comparisons**						
Incasive sham acupuncture group (*n* = 107)		18 sessions of acupuncture at non-acupoints				
Waiting-list group (*n* = 107)		18 sessions of treatment at the end of the trial				
**Outcomes**						
24-h blood pressure		x		x	x	x
Expectation of acupuncture		x				
SF- 36		x		x		
Response to treatment				x		
**Participants safety**						
Laboratory test		x				
Adverse events		x	x	x	x	x

SAS, Self-Rating Anxiety Scale; SDS, Self-Rating Depression Scale; SF-36, MOS item Short Form health survey.

### Randomization

Randomization is conducted by the third party (the Brightech Magnsoft Data Services Company) using text messages or a website. If the randomization succeeds, the investigators will receive an email confirmation. The randomization sequence (blocked, stratified for centers) is generated through the randomization module of the synthesized management platform of the Brightech Magnsoft Data Services Company (block length is unknown to centers). This procedure ensures each eligible patient having an equal probability of being assigned to four different groups, which will not be influenced by the researchers.

### Participants

Patients who meet the inclusion criteria will be included in this study. The criteria are as follows: (1) men or women aged between 40 and 75 years,; (2) participants meet the diagnostic criteria of mild hypertension according to JNC-7 [12] and 2010 Chinese guidelines for the management of hypertension [5](diagnosed as mild hypertension systolic blood pressure (SBP) 140–159 and/or diastolic blood pressure (DBP) 90–99 ,according to WHO/ISH criteria stage I); (3) patients who are initially diagnosed as mild hypertension or have been diagnosed as mild hypertension before, without taking any antihypertensive drugs; (4) patients who are diagnosed as Jue-yin syndrome (yin-deficiency accompany with hyperactivity of yang) or Yang-ming syndrome (obstruction of phlegm and dampness) according to meridian syndrome differentiation; (5) patients who are willing to comply with our study protocol; (6) participants agree to sign informed consent form.

The exclusion criteria are as follows: (1) patients who have been diagnosed with secondary hypertension [13] or malignant hypertension (for example, Cushing’s syndrome, coarctation of the aorta, pheochromocytoma, renal parenchymal disease, primary aldosteronism, renovascular hypertension, obstructive sleep apnea, drug-induced hypertension, and so on); (2) patients accompanied with other severe medical conditions (for example, endocrine disorders, cardiovascular disease, digestive disease, hepatic dysfunction, cerebral vascular disease, renal disease, hematologic disease,and so on); (3) patients with a chronic disease (for example, epilepsy, severe depression, or anxiety (SAS ≥70, SDS ≥70), psychosis, allergic constitution, accompany with any infection), pregnant women or women in lactation or women of childbearing potential planning to conceive in the next 6 months; (4) patients who currently participate in another clinical trial; (5) patients who had been treated with acupuncture during the previous 3 months. If one of the criteria mentioned above is matched, the participant will be excluded.

During the whole study period, participants will not be hospitalized, taking into account: (1) we only recruit patients with mild essential hypertension who went off hypertensive drugs and without complications; (2) we could observe the blood pressure of participants under regular activities; (3) patients with mild essential hypertension are not appropriate to recruit for inpatient treatment.

#### ***Recruitment strategies***

We will apply two strategies to recruit participants with mild hypertension. First, we recruit participants from outpatient clinics of the participated hospitals. Second, we employ three kinds of advertisements to attract patients to join our study. We will distribute printed recruitment posters in the participated hospitals and the communities nearby. Meanwhile we will publish advertisement in local newspaper and broadcast television advertisements on the local channel.

#### ***Intervention***

##### 

**Rationale for selection of acupoints** According to TCM theory, hypertension pertains to the coverage of vertigo and headache. Based on previous survey and expert consultation, Jue-yin syndrome (yin deficiency with yang hyperactivity) and Yang-ming syndrome (the obstruction of phlegm and dampness) are the most common syndromes in mild hypertension. Therefore, in accordance with meridian syndrome differentiation, we select acupoints on the Yang-ming, Jue-yin, and Shao-yin meridians. Hence, it is reasonable that the acupoints on the affected meridians would be most effective at treating mild hypertensive patients. Meanwhile we also choose acupoints on the non-affected meridians (Gallbladder, San-jiao, and Spleen meridians) which were reported to be useful to decrease blood pressure according to our previous literature research.

In our trial, there are four groups: (1) affected meridian group;(2) non-affected meridian group;(3) sham acupuncture group;and (4) waiting-list group (Table 2). Apart from the waiting-list group, the other patients assigned to the three acupuncture groups will each receive 18 sessions of acupuncture treatment.

1. Affected meridian acupuncture (AMA)

**Figure 2:**
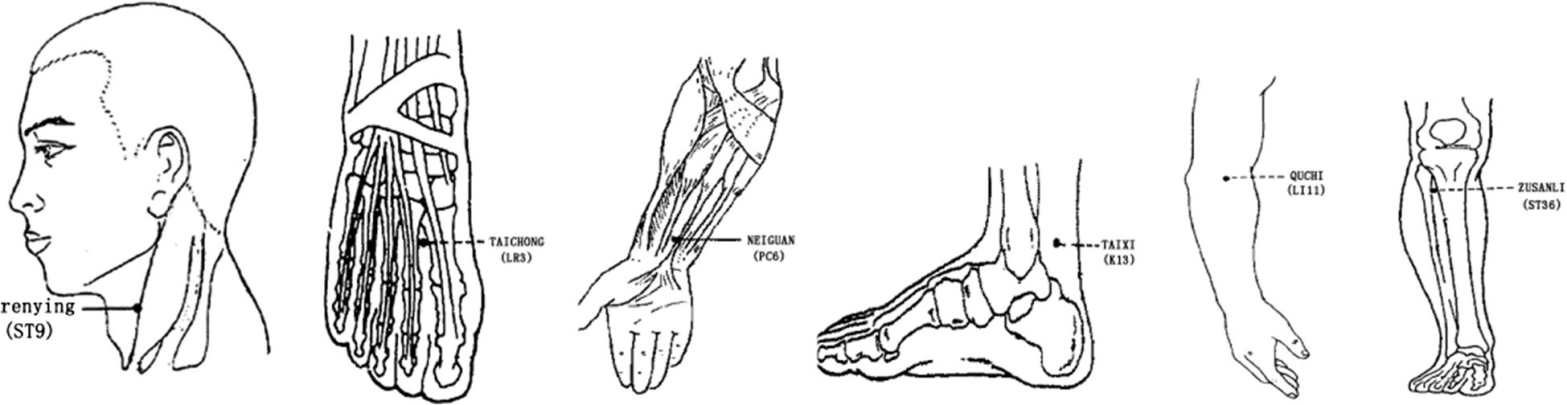
Locations of acupoints: affected meridian acupuncture group (AMA).

Participants in the affected meridian group (Figure 2) are treated according to two syndromes (Jue-yin syndrome and Yang-ming syndrome). Jue-yin syndrome: taichong (LR3), renying (ST9), taixi (KI3), and neiguan (PC6) are punctured with filiform needles bilaterally. Taichong (LR3) is punctured perpendicularly 0.5-1.0 cun. Renying (ST9) is punctured perpendicularly 0.3-0.8 cun, paying attention to avoiding the carotid artery. Taixi (KI3) is punctured perpendicularly 0.5-1.5 cun. Neiguan (PC6) is punctured perpendicularly 0.5-1.0 cun. Yang-ming syndrome: taichong (LR3), renying (ST9), zusanli (ST36), and quchi (LI11) are punctured with filiform needles bilaterally. Taichong (LR3) is punctured perpendicularly 0.5-1.0 cun. Renying (ST9) is punctured perpendicularly 0.3-0.8 cun, paying attention to avoiding the carotid artery. Zusanli (ST36) is punctured perpendicularly 1.0-2.0 cun. Quchi (LI11) is punctured perpendicularly 1.0-1.5 cun.

After insertion, all the acupoints will be manually stimulated by lifting and thrusting associated with twirling and rotating the needle, to elicit 'de-qi’. Jue-yin syndrome and Yang-ming syndrome will be identified by the practitioner according to the diagnostic characteristic variables of TCM (Table 3).

2. Non-affected meridian acupuncture (NMA)

**Figure 3:**
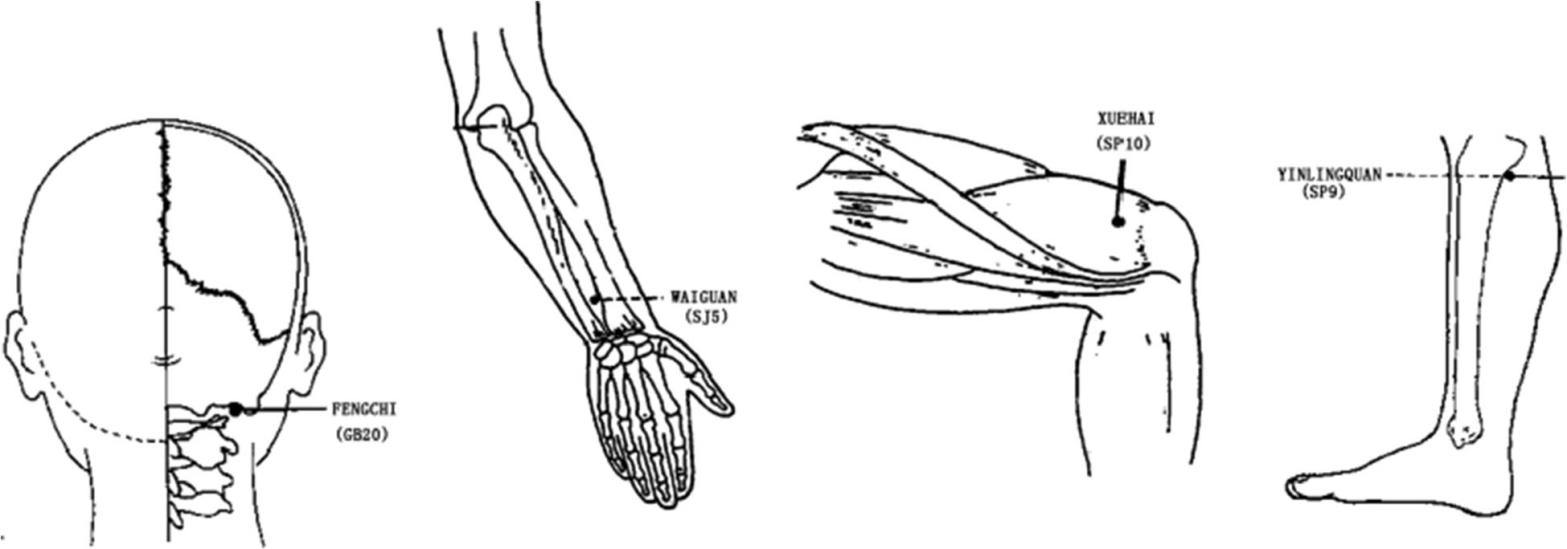
Locations of acupoints: non-affected meridian acupuncture group (NMA).

Fengchi (GB20), waiguan(SJ5), yinlingquan(SP9), and xuehai (SP10) are punctured by filiform needles bilaterally (Figure 3). Fengchi (GB20) is punctured obliquely 0.8-1.2cun, with the tip of the needle pointing to the apex of nose. Waiguan (SJ5) is punctured perpendicularly 0.5-1.0 cun. Yinlingquan (SP9) is punctured perpendicularly 1.0-2.0 cun. Xuehai (SP10) is punctured perpendicularly 1.0-1.5cun. All the acupoint locations are in accordance with the WHO Guideline for Acupuncture Point Locations [14].

After insertion, all the acupoints will be manually stimulated by lifting and thrusting associated with twirling and rotating the needle, to search for 'de-qi’.

3. Invasive sham acupuncture (ISA)

**Figure 4:**
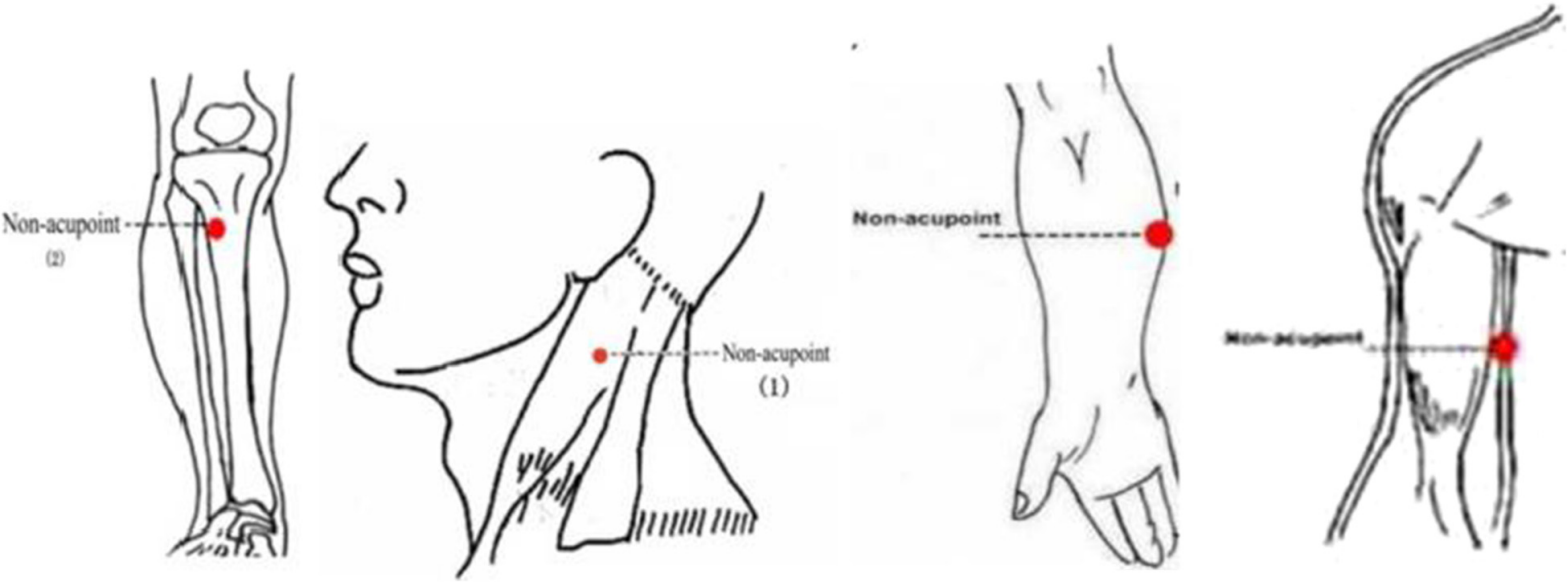
Locations of non-acupoints: invasive sham acupuncture group (ISA).

Sham acupoints (Figure 4):

(1) The edge of the tibia (1–2 cm lateral and horizontal to the zusanli (ST36))

(1) Halfway between the tip of the elbow and the axilla

(1) On the ulnar side of the arm, halfway between the epicondylus medialis of the humerus and the ulnar side of the wrist

(1) 2 cm superior to fu tu (LI18)

We use pre-validated sham acupoints [15]. After the insertion, we will not use any manipulationand 'de-qi’ sensation is not needed.

4. Waiting-list (WL)

Participants in the WL group will not be treated throughout 13-week observation period. And they will be required to maintain their current lifestyle, including diet and exercise. At the end of trial, if the participant would like to be treated with acupuncture, we will offer acupuncture treatment three times weekly for 6 weeks.

**Table 2:** Details of acupuncture protocol

AMA	Jue-yin syndrome	Taichong (LR3), renying(ST9), taixi(KI3), neiguan(PC6)
	Yan-ming syndrome	Taichong (LR3), renying(ST9), zusanli(ST36), quchi(LI11)
NMA		Fengchi(GB20), waiguan(SJ5), yinlingquan(SP9), xuehai(SP10)
ISA		1. The edge of the tibia (1–2 cm lateral and horizontal to the zusanli (ST36))
		2. Halfway between the tip of the elbow and the axilla
		3. On the ulnar side of the arm, halfway between the epicondylus medialis of the humerus and the ulnar side of the wrist
		4. 2 cm superior to futu(LI18)

AMA, Affected meridian acupuncture group; ISA, Invasive sham acupuncture group; NMA, Non-affected meridian acupuncture group.

**Table 3:** Diagnostic characteristic variables of TCM

	**Yin deficiency with yang hyperactivity**	**Obstruction of phlegm and dampness**
Pulse	Wiry, thin,fast	Slippery and soggy
Tongue	Red with no coating, peeled	Greasy coating, swollen
Other primary symptoms	Vertigo, headache, malar flush, both knee and loin feel sore and weak, dysphora in chest, palms, and soles, irritable, dry mouth, dry eyes, palpitation, insomnia, poor memory	Heavy head and or body, muddled thinking, overweight, vertigo, palpitation, poor appetite, nausea, stuffy feeling in the chest and/or epigastrum, heavy frontal headache, insomnia
Secondary symptoms	Concentrated dark urine, dry stools, blurred vision, numbness and tremors of limbs, tinnitus	Tinnitus, numbness in the limbs, loose stools

During the whole study period, all the participants are not allowed to take any medical treatment for lowering blood pressure; drugs, herbs, and physical therapy are forbidden. If the participants suffer from other diseases and need to be treated with drugs,they have to record the drugs they take, and measure their blood pressure after drugs, all of which will be recorded in the case report forms.

In each session, acupuncture is applied bilaterally. After insertion, apart from the acupoints around the neck (ST9, GB20, non-acupoints4), six auxiliary needles will be punctured at 2 mm lateral to each acupoint or non-acupoint. As to the auxiliary needle, we will not do any manipulation. We use transcutaneous electric acupoints stimulation (HANS; Han’s acupoints nerve stimulator, HANS-200, Nanjing, China) to stimulate the acupoints and non-acupoints. Except for the acupoints around the neck (ST9, GB20, non-acupoints 4), the other acupoints or non-acupoints and their auxiliary needles are connected with electricity by HANS for 30 min, the frequency is 2HZ, and the intensity is confined to 2 mA. It had been demonstrated that 30 min of low-frequency electro-acupuncture activates opioid receptors and provides a therapeutic effect on hypertension [16,17].

The treatment will be performed after sterilizing the skin with 75% alcohol on the areas where the needles will be inserted, and with the patient lying in the supine position. We use two types of Hwato disposable steel needles (Suzhou Hua Tuo Medical Instuments, Suzhou, China): length 25–40 mm, diameter 0.25 mm; and length 13 mm, diameter 0.18 mm. All the groups but the patients in waiting-list group are asked to retain the needles for 30 min.

Apart from waiting-list group, the patients from the other groups will receive 18 sessions of acupuncture treatment over 6 weeks, three times per week. During the whole observation period, no patients are allowed to take antihypertensive drugs.

#### ***Outcome measurement***

##### 

**Primary endpoint** The primary outcome measurement in this study is the change in average 24-hSBP and DBP before randomization and 6 weeks after randomization. Ambulatory mean blood pressure (ABPM) is superior to clinic BP in the prediction of cardiovascular morbidity and mortality [18,19]. Because ABPM exerts a different predictive effect on the cardiac and cerebrovascular complications [20] we selected ABPM as our primary endpoint.

##### 

**Secondary outcome measures** Ambulatory pulse pressure (APP) [21] is a well-established marker of cardiovascular risk. We choose APP as one of the secondary endpoints to evaluate the effect of acupuncture for hypertension. There is a prospective study [22] that shows that both high ABPM values and abnormal daytime/night-time BP profiles are associated with later occurrence of ischemic cerebrovascular and coronary events independently of casual BP values and other cardiovascular risk factors. It is therefore necessary to observe the daytime/night-time BP of hypertensive patients.

Changes in patients’ health-related quality of life, according to the SF-36 (Medical Outcomes Study 36-Item Short Form) questionnaire Chinese version is another secondary outcome measure [23]. The validity and reliability of this scale have proven in previous studies [24,25]. The scale contains eight dimensions (physical function, role physical, bodily pain, general health, vitality, social function, role emotional, mental health) and two summary components (physical and mental), measured on a scale of 0 to 100. Lower scores indicate a poorer quality of life. SF-36 investigates at 0 day and 6 weeks after randomization.

Response to the treatment will be measured at the end of treatment, using a five-point Likert self-administered scale:'Compared with pre-treatment, to what extend did you think your hypertensive symptoms change?’1, absence of all symptoms; 2, significantly improved; 3, moderately improved; 4, not changed; 5, deteriorated. 'After treatment, how do you evaluate the effect of acupuncture for hypertension?’ 1, no effect(0%); 2, a little effect(25%); 3, moderate effect(50%); 4, a significant effect (75%); 5, complete response(100%). The patients fill in this questionnaire at the end of the treatment.

Expectation of the treatment will be measured by a self-administered questionnaire. With seven items as follows: (1) In the coming year, how will you think your disease will be relieved? (significantly(≥75%); moderately(approximately 50% to 75%); a little(approximately 25% to 50%); no change(0% to 25%)); (2) How effective do you think acupuncture is for hypertension? (significant(≥75%); moderate(approximately 50% to 75%); a little(approximately 25% to 50%); no change(approximately 0% to 25%)); (3) Which treatment options do you prefer to take as your treatment for hypertension? (acupuncture and moxibustion; herbs; Western medicine; others); (4) How familiar are you with acupuncture and moxibustion? (very familiar; so-so; a little; not at all); (5) By what means did you get to know about acupuncture and moxibustion? (friends and relatives; media reports; medical work; others); (6) How did acupuncture and moxibustion work in your memory? (excellent; good; so-so; not at all); (7) During the treatment, are you afraid of the pain or uncomfortable sensation caused by acupuncture and moxibustion? (very much; a little; never think about it; feel comfortable). The subjects will mark the best answer for each of the questions. The assessment will be performed at day 0 after randomization.

Before randomization, a special pattern identification questionnaire was developed to differentiate which syndrome the subjects pertaining to.

#### ***Blood pressure measurement***

Ambulatory blood pressure measurements are performed with an oscillometric device (A&D Co.Ltd., Japan TM-2430). During a 24-h monitoring period, the device takes 76 measurements. Daytime is defined as 08:00–22:00. The measurement during the daytime will be taken every 15 min. Night-time is defined as 22:00–08:00. The measurement during the night-time will be taken every 30 min. The 24-h ambulatory blood pressure monitoring measures at baseline, 6 weeks, 9 weeks, and 12 weeks after randomization. Clinical blood pressure measurements will be taken before and after each acupuncture session with Automatic Upper Arm Blood Pressure Monitor (Omron HEM 7200, Liaoning, China).

#### ***Participant safety***

Before randomization, all participants will undergo routine tests of blood, urine, stool sample, electrocardiogram examination, Doppler echocardiography, carotid ultrasonography, as well as blood biochemical tests (AST, ALT, BUN, Scr, fasting glucose, triglyceride, HDL cholesterol, LDL cholesterol, albumin/creatinine.). These tests will help identify and exclude participants with serious illnesses of the heart, liver, and kidneys, or other severe diseases.

Adverse events are defined as any unexpected or uncomfortable signs, symptoms, or diseases, regardless of the intervention. These adverse events include bleeding, hematoma, fainting, serious pain, and local infection. If any adverse events happen during the entire observation period, all the details should be documented in the case report form.

We have designed a case reported form containing a variety of information, and this will be completed by the corresponding researchers at each center. At each center, the obtained information will be recorded on an electronic database, for subsequent statistical analysis. During the observation period, if there are any drop-outs or withdrawals, the investigator must find out their reasons and record them in the case report form. The patients’ compliance during the 6 weeks of treatment and 6 weeks of follow-up will also be documented.

#### ***Sample size calculation and statistical analysis***

According to the previous literature [26], the average 24-hSBP of hypertensive patients rose 1.6 mmHg after being treated by sham acupuncture, while the average 24-hSBP of hypertensive patients lowered 5.4 mmHg after being treated with verum acupuncture. Therefore, the adjusted mean difference between the two groups was 7 mmHg. In this study, we anticipated an 12 mmHg improvement by affected meridian acupuncture; 10 mmHg by non-affected meridian acupuncture; 3 mmHg by sham acupuncture; and 0 mmHg in the waiting-list group. The standard deviation is set as 20. We calculated this using 90% power at a 5% significance level with G*Power (version 3.1.2, Franz Faul, Universität Kiel, Germany), and the result showed an estimated 93 patients per group. Considering a drop-out rate of 15%, we plan to enroll 107 patients per group, 428 patients in total for the four different groups.

Statistical analysis protocol was designed by the Brightech-Magnsoft Data Services Company and principal investigators. Analysis of all data in this trial will be performed by the Brightech-Magnsoft Data Services Company. The data will be analyzed with SAS 9.1 and SPSS13.0 software packages. All analyses will be on the basis of the intention-to-treat (ITT) population and per-protocol (PP) population. The result of the ITT analysis will be compared with that of the PP analysis to determine whether the results are consistent. Missing data will be filled by Last Observation Carry Forward rules.

Demographic characteristics and other baseline values will be described using descriptive statistics for each group. For the primary outcome, we will run a comparison between pooled acupuncture groups and waiting list control group to figure out whether acupuncture is more effective for this condition. We will then run another comparison between the pooled acupuncture groups and sham acupuncture group to find out whether acupuncture is more efficacious. Finally, a comparison between acupuncture groups will be run to see if selecting points along meridians was a better treatment option. The above analysis will be performed using the ANCOVA model. Multiple comparisons will be adjusted according to the Bonferroni correction method.

The repeated measures analysis will be used in the different time point assessments for the secondary outcomes. The Kruskal-Wallis test will be employed in the analysis of skewed distribution data. There is a designated acupuncturist in each center to all the acupuncture treatment, which probably introduces clustering effect in this trial. Therefore, we calculated the intra-cluster correlation coefficient from the results of this trial and report the coefficient.

## Discussion

Acupoints are the regions where qi and blood from the viscera and meridians effuse and infuse beneath the body surface; the meridians and collaterals internally pertain to viscera and externally connect with the extremities. Therefore, stimulating the acupoints on the affected meridians can produce specific effects on regulating the corresponding organs, and the specificity of acupoint effect is the important basis for this regulation effect [27,28]. However, the specificity of acupoint effect on the affected meridians, continues to be a conflicting theoratical issue in acupuncture field [29,30]. In recent years, much research on acupoint specificity has been launched, but the resultsare still inconclusive. There is a small sample study of 41 randomized hypertensive patients into two groups (one gave verum acupuncture, and the other received non-penetrating sham acupuncture). After 17 treatment sessions, the verum acupuncture group showed a significant decrease in blood pressure [31]. Similar results had been found in another study, which discovered that unlike sham acupuncture, after 6weeks of treatment with acupuncture according to TCM, the verum acupuncture significantly decreased mean 24-h ambulatory blood pressure [26]. On the other hand, another study of a higher quality obtained negative results on comparing active acupuncture with invasive sham acupuncture. They founded that active acupuncture provided no greater benefit than invasive sham acupuncture in reducing SBP or DBP [32].

Therefore, we launch this trial by means of multiple comparisons of four different arms (affected meridian acupuncture, non-affected meridian acupuncture, invasive sham acupuncture, and waiting-list groups) to aim to clarify this issue. First, we compare the efficacy of acupoints on the affected meridian with the waiting-list group who do not receive any intervention during the whole observation period. Second, we compare this with the invasive sham acupuncture group. We then compare the acupoints on the non-affected meridians. We believe that affected meridians based on syndrome differentiation acupuncture are capable of lowering the elevated blood pressure of hypertensive patients, of which effect is better than non-affected meridian acupuncture, and to a greater degree than the sham acupuncture and waiting-list groups. It can enhance health-related quality of life. Furthermore, as a treatment option for hypertension, it caused less adverse side effects. Through this trial, we attempt to demonstrate the real effectiveness of acupuncture for hypertension, and illustrate the specific effect of acupoints on the affected meridian.

Design highlights: first, unlike previous trials, we only include mild hypertensive patients without serious complications as it is safe for all subjects to go off hypertensive drugs and only be treated with acupuncture. We also rule out the influence from hypertensive drugs. Besides, by focusing on mild hypertension [33], we can cut down the compounding effect from different severities of hypertension. Second, we use electro-stimulation in the acupuncture groups aiming to ensure the stimulations between groups and individualsare equal. Third, in order to ensure the quality of this trial, we require that all the participating acupuncturists have at least 5 years of training and 5 years of practical experience. Before the trial, they will take a training course which includes the study protocol, methods for acupuncture treatment, and operating demonstration for this trial. After the course, they need to take an exam about the training course to be qualified for this trial. Meanwhile, we will designate special clinical monitors to check the progress of the trial and audit the accuracy and validity of the original data at each participating hospital once a month. Fourth, in our trial, a first ever large sample of patients will be recruited, which will make the results more powerful and convincing. Moreover, our design has a good understanding of theory of TCM and acupuncture.

One important limitation that may be present in this study is that it is an open-label trial. We design a waiting-list group as a blank controlled group. It is impossible to execute a blinding method. In order to overcome this problem, the acupuncturist and the outcome assessors are different, with respect to making the outcome assessment objectively. We also prevent the investigators and statisticians from randomization, to make sure all of them are blind to allocation. Among the three acupuncture groups, we conceal the patients from assignation so the patients do not know which acupuncture group they are allocated in.

This is a large scale, multicenter, randomized, controlled trial, and utilizes high quality methodologies. It may provide evidence for the effectiveness of acupuncture as a treatment for mild hypertension and confirm the existence of special effect of acupoints on affected meridians based on meridian syndrome differentiation.

## Trial status

The first participant was included on 30 September 2012, and this article was submitted on 30 June. To date, 184 participants have been recruited.

## Competing interests

The authors declare that they have no competing interests.

## Authors’ contributions

JL, HZ, YL, LZ, DHL, XL, JC, ZY, and FRL participated in the conception and design of the trial. Planning the analysis of the data and drafting the manuscript. XRC RHW, JC, JS, and YLH participated in data collection and in charge of recruitment and treatment of patients in each center. All authors read and approved the final manuscript.

## References

[B1] Kearney PM Whelton M Reynolds K Muntner P Whelton PK He J Global burden of hypertension: analysis of worldwide data Lancet 2005 365 217 223 15652604 10.1016/S0140-6736(05)17741-1

[B2] Ong KL Cheung BM Man YB Lau CP Lam KS Prevalence, awareness, treatment, and control of hypertension among United States adults 1999–2004 Hypertension 2007 49 69 75 10.1161/01.HYP.0000252676.46043.18 17159087

[B3] Lloyd-Jones D Adams R Carnethon M De Simone G Ferguson TB Flegal K Ford E Furie K Go A Greenlund K Heart disease and stroke statistics–2009 update a report from the American heart association statistics committee and stroke statistics subcommittee Circulation 2009 119 480 486 19171871 10.1161/CIRCULATIONAHA.108.191259

[B4] Li LM Rao KQ Kong LZ Yao CH Xiang HD Zhai FY Ma GS Yang XG A description on the Chinese national nutrition and health survey in 2002 Chin J Epidemiol 2005 26 478 484 16334996

[B5] Liu LS Chinese guidelines for management of hypertension Chin J Hypertens 2010 2011 19 701 708

[B6] Wang J-G Li Y Characteristics of hypertension in the Chinese population Curr Hypertens Rep 2012 14 410 415 10.1007/s11906-012-0288-1 22843493

[B7] Zhai Y HU JP Kong LZ Zhao WH Chen CM **Economic burden of coronary heart disease and stroke attributable to hypertension in China** Chin J Epidemiol 2006 27 744 747 17299955

[B8] Diao D Wright JM Cundiff DK Gueyffier F Pharmacotherapy for mild hypertension Cochrane Database Syst Rev 2012 8 CD006742 22895954 10.1002/14651858.CD006742.pub2 PMC8985074

[B9] Lin MC Nahin R Gershwin ME Longhurst JC Wu KK State of complementary and alternative medicine in cardiovascular, lung, and blood research: executive summary of a workshop Circulation 2001 103 2038 2041 10.1161/01.CIR.103.16.2038 11319191

[B10] Richter A Herlitz J Hjalmarson A Effect of acupuncture in patients with angina pectoris Eur Heart J 1991 12 175 178 10.1093/eurheartj/12.suppl_D.175 2044550

[B11] Zhou W Longhurst JC Neuroendocrine mechanisms of acupuncture in the treatment of hypertension Evid Based Complement Altern Med 2011 2012 878673 10.1155/2012/878673 PMC3246758 22216059

[B12] Chobanian AV Bakris GL Black HR Cushman WC Green LA Izzo JL Jones DW Materson BJ Oparil S Wright JT Seventh report of the joint national committee on prevention, detection, evaluation, and treatment of high blood pressure Hypertension 2003 42 1206 1252 10.1161/01.HYP.0000107251.49515.c2 14656957

[B13] Mancia G De Backer G Dominiczak A Cifkova R Fagard R Germano G Grassi G Heagerty AM Kjeldsen SE Laurent S Guidelines for the management of arterial hypertension the task force for the management of arterial hypertension of the European society of hypertension (ESH) and of the European society of cardiology (ESC) Eur Heart J 2007 2007 28 1462 1536 17562668 10.1093/eurheartj/ehm236

[B14] WHO Regional Office for the Western Pacific WHO standard acupuncture point locations in the western pacific region 2008 Manila: WHO

[B15] Li Y Zheng H Witt CM Roll S Yu S-g Yan J Sun G-j Zhao L Huang W-j Chang X Acupuncture for migraine prophylaxis: a randomized controlled trial Can Med Assoc J 2012 184 401 410 10.1503/cmaj.110551 22231691 PMC3291669

[B16] Zhou W Fu L-W Tjen-A-Looi SC Li P Longhurst JC Afferent mechanisms underlying stimulation modality-related modulation of acupuncture-related cardiovascular responses J Appl Physiol 2005 98 872 880 15531558 10.1152/japplphysiol.01079.2004

[B17] Zhou W Tjen-A-Looi SC Longhurst JC Brain stem mechanisms underlying acupuncture modality-related modulation of cardiovascular responses in rats J Appl Physiol 2005 99 851 860 10.1152/japplphysiol.01365.2004 15817715

[B18] Health Quality Ontario Twenty-four-hour ambulatory blood pressure monitoring in hypertension: an evidence-based analysis Ont Health Technol Assess Ser 2012 12 1 65 PMC3377518 23074425

[B19] Clement DL De Buyzere ML De Bacquer DA de Leeuw PW Duprez DA Fagard RH Gheeraert PJ Missault LH Braun JJ Six RO Prognostic value of ambulatory blood-pressure recordings in patients with treated hypertension N Engl J Med 2003 348 2407 2415 10.1056/NEJMoa022273 12802026

[B20] Verdecchia P Schillaci G Reboldi G Franklin SS Porcellati C Different prognostic impact of 24-hour mean blood pressure and pulse pressure on stroke and coronary artery disease in essential hypertension Circulation 2001 103 2579 2584 10.1161/01.CIR.103.21.2579 11382727

[B21] Verdecchia P Schillaci G Borgioni C Ciucci A Pede S Porcellati C Ambulatory pulse pressure a potent predictor of total cardiovascular risk in hypertension Hypertension 1998 32 983 988 10.1161/01.HYP.32.6.983 9856961

[B22] Bastos J Bertoquini S Silva J Polonia J Relationship between ambulatory blood pressure monitoring values and future occurrence of ischemic cerebrovascular and coronary events in hypertensive patients Rev Port Cardiol 2006 25 305 16789404

[B23] Fang JQ Quality of life measurement method and application 2000 Peking University Medical Press

[B24] Wang CH Fang JQ Tang XL Zhang CZ Lu YB Meng Q Gao L The effect evaluation on measuring quality of life in patients with liver cancer by SF-36 scale Tumor 2005 25 492 494

[B25] Wang CH Fang JQ Shi ML Chen LY Zhang YZ A comparative study on measuring quality of life for drug addicts by SF-36 scale Chin J Behav Med Sci 1998 7 260 261

[B26] Flachskampf FA Gallasch J Gefeller O Gan J Mao J Pfahlberg AB Wortmann A Klinghammer L Pflederer W Daniel WG Randomized trial of acupuncture to lower blood pressure Circulation 2007 115 3121 3129 10.1161/CIRCULATIONAHA.106.661140 17548730

[B27] Liang F Fang Z Zhao L Tang Y Specificity of acupoint effects and its fundamental laws Zhongguo Zhen Jiu 2009 29 129 19391538

[B28] Ding XY She YF Ma LX Zhu J Three basic characteristics of specificity of acupoint effect China J TradiChin Medic Pharma 2010 25 1356 1359

[B29] Choi EM Jiang F Longhurst JC Point specificity in acupuncture Chin Med 2012 7 1 5 10.1186/1749-8546-7-1 22373514 PMC3311034

[B30] Zhao L Chen J Liu C-Z Li Y Cai D-J Tang Y Yang J Liang F-R A review of acupoint specificity research in china: status quo and prospects Evid Based Complement Alter Medic 2012 2012 543943 10.1155/2012/543943 PMC3518822 23243454

[B31] Yin C Seo B Park H-J Cho M Jung W Choue R Kim C Park H-K Lee H Koh H Acupuncture, a promising adjunctive therapy for essential hypertension: a double-blind, randomized, controlled trial Neurol Res 2007 Suppl 1 98 103 10.1179/016164107X172220 17359649

[B32] Kalish LA Buczynski B Connell P Gemmel A Goertz C Macklin EA Pian-Smith M Stevens S Thompson J Valaskatgis P Stop hypertension with the acupuncture research program (SHARP): clinical trial design and screening results Control Clin Trials 2004 25 76 103 10.1016/j.cct.2003.08.006 14980754

[B33] The National Heart, Lung, and Blood Institute Working Group on Future Directions in Hypertension Treatment Trials Major clinical trials of hypertension: what should be done next? Hypertension 2005 46 1 615911739

